# ERCP in infants, children, and adolescents—Different roles of the methods in different age groups

**DOI:** 10.1371/journal.pone.0210805

**Published:** 2019-01-17

**Authors:** Radan Keil, Jiří Drábek, Jindra Lochmannová, Jan Šťovíček, Petra Koptová, Martin Wasserbauer, Barbora Frýbová, Jiří Šnajdauf, Jan Matouš, Radana Kotalová, Michal Rygl, Štěpán Hlava

**Affiliations:** 1 Department of Internal Medicine, Charles University, 2^nd^ Faculty of Medicine, University Hospital Motol, Prague, Czech Republic; 2 Department of Pediatric Surgery, Charles University, 2^nd^ Faculty of Medicine, University Hospital Motol, Prague, Czech Republic; 3 2^nd^ Department of Internal Medicine Charles University, 3^rd^ Faculty of Medicine, FNKV, Prague, Czech Republic; 4 Department of Pediatry, Charles University, 2^nd^ Faculty of Medicine, University Hospital Motol, Prague, Czech Republic; Medizinische Fakultat der RWTH Aachen, GERMANY

## Abstract

**Background:**

Endoscopic retrograde cholangiopancreatography (ERCP) is seldom used in children, and published series have limited numbers of pediatric patients. The aim of this retrospective observational study was to assess the efficacy and safety of pediatric ERCP in a large group of children.

**Methods:**

Data were evaluated from 626 children with biliopancreatic disorders admitted to University Hospital Motol, Prague, between January 1999 and January 2018. Clinical data were obtained by retrospective evaluation of our database of pediatric ERCP procedures and from clinical records.

**Results:**

We performed 856 ERCPs on 626 pediatric patients; of these procedures, 59% were therapeutic and 41% were diagnostic. We achieved 96% technical success. Indications for ERCP and pathological findings differed in different age groups. The main role of ERCP was in excluding biliary atresia in those aged less than one year. In children aged 1 to 6 years, the most frequent diagnoses were choledochal cyst followed by choledocholithiasis. In children aged 7 to 12 years and 13 to 19 years, the most frequent diagnoses were choledocholithiasis followed by pancreatic pathology. The overall complication rate found in this study was similar to rates observed in adult populations.

**Conclusions:**

Our study shows the efficacy and safety of diagnostic and therapeutic ERCP in a large series of infants and children with technical success and complication rates comparable to those in adults. Our data show that ERCP had different roles in different age groups of children.

## Introduction

Endoscopic retrograde cholangiopancreatography (ERCP) is a therapeutic and diagnostic technique used routinely in adults. Over the past four decades, there have been technological advancements in noninvasive cross-sectional imaging modalities including the development of magnetic resonance cholangiopancreatography (MRCP). MRCP replaced ERCP as a diagnostic method, but ERCP continues to be a preferred modality for therapeutic interventions in the pancreaticobiliary system. [1]

The indications for ERCP in children are relatively rare and published series have a limited number of pediatric patients.[2–9] Therefore, the method is not commonly used for biliopancreatic disorders. Its conservative use is a result of lack of experience, lack of data about the safety of this method, and its infrequent indication in children and infants.[10] On the other hand, ERCP is used more frequently to investigate and treat pediatric populations in the USA.

A report utilizing a US healthcare administrative database showed that between 2000 and 2009, the use of ERCP in the pediatric population grew steadily, approaching 7000 cases per year by the end of the decade. [11] In contrast, the total number of patients examined remains small compared to the adult literature, and questions remain regarding the appropriate utilization of ERCP in children.[1]

The aim of this retrospective observational study was to characterize the indications for ERCP in a pediatric population and differentiate the use of ERCP in infants, children, and adolescents. We wanted to assess the efficacy and safety of ERCP procedures in pediatric patients performed by gastroenterologists, who usually treat adults, at a tertiary care center. We also wanted to demonstrate different technical equipment, different indications for the method, different frequencies of diagnostic and therapeutic procedures in each age group, and the effect on patients.

We retrospectively analyzed ERCP, ERCP-related interventions, and procedure-related complications in infants and children at our high-volume endoscopy department.

## Materials and methods

In our endoscopic center, we perform 1600–1800 ERCPs in adult patients every year and provide services for children and infants from the area, which includes 5 million inhabitants. Care for these children is organized with close cooperation between adult gastroenterologists, pediatricians and pediatric surgeons.

Between January 1999 and January 2018, 626 pediatric patients with biliopancreatic disorders were evaluated. There were 294 boys and 332 girls aged from 12 days to 17 years and 6 months. Every patient aged less than 19 years referred to our endoscopic center during the years 1999–2018 was included in this observational, retrospective study. All ERCP procedures in children aged less than 2 years were performed by two endoscopists. Procedures in children aged from 2 to 18 years were performed by four endoscopists. All endoscopists had 10 or more years of experience and have performed 3000–4000 ERCPs in adult patients.

Clinical data were obtained by retrospective evaluation from our database of pediatric ERCP procedures and from clinical records. Indications were based on clinical history, physical examination, liver function tests, serum amylase and lipase levels, abdominal ultrasound, computed tomography, and MRCP findings. Before performing ERCP, informed written consent was obtained from all the parents of our patients. Study was approved by Ethic Committee of the University Hospital Motol and 2^nd^ Faculty of Medicine, Charles University in Prague, Reference No: EK-1100/18.

All examinations were carried out on a fluoroscopic table under general anesthesia, with continuous monitoring of vital functions. For children aged less than 12 months or weighing less than 12 kg, it was necessary to use only the pediatric duodenoscope (Olympus PJF), with a 7.5 mm outer diameter and 2-mm working channel. The lowest body weight in our cohort was 1.4 kg. For cannulation, ultra-thin handmade cannulas were used.

The therapeutic role of ERCP was limited in children aged less than one year because the pediatric duodenoscope from Olympus should not be used for papilla sphincterotomy; therefore, biliary drainage and pancreatic drainage was resolved by insertion of a biliary stent without papilla sphincterotomy. For insertion of stents 5 fr in diameter it was necessary to use a double lumen sphincterotome with an outer diameter of 1.75 mm (Medi-Globe RotaCut GSP-21-17-020) and a guide wire with an outer diameter of 0,53 mm (Cook METII-21-480 Tracer Metro Direct Wire Guide).

**Fig 1:**
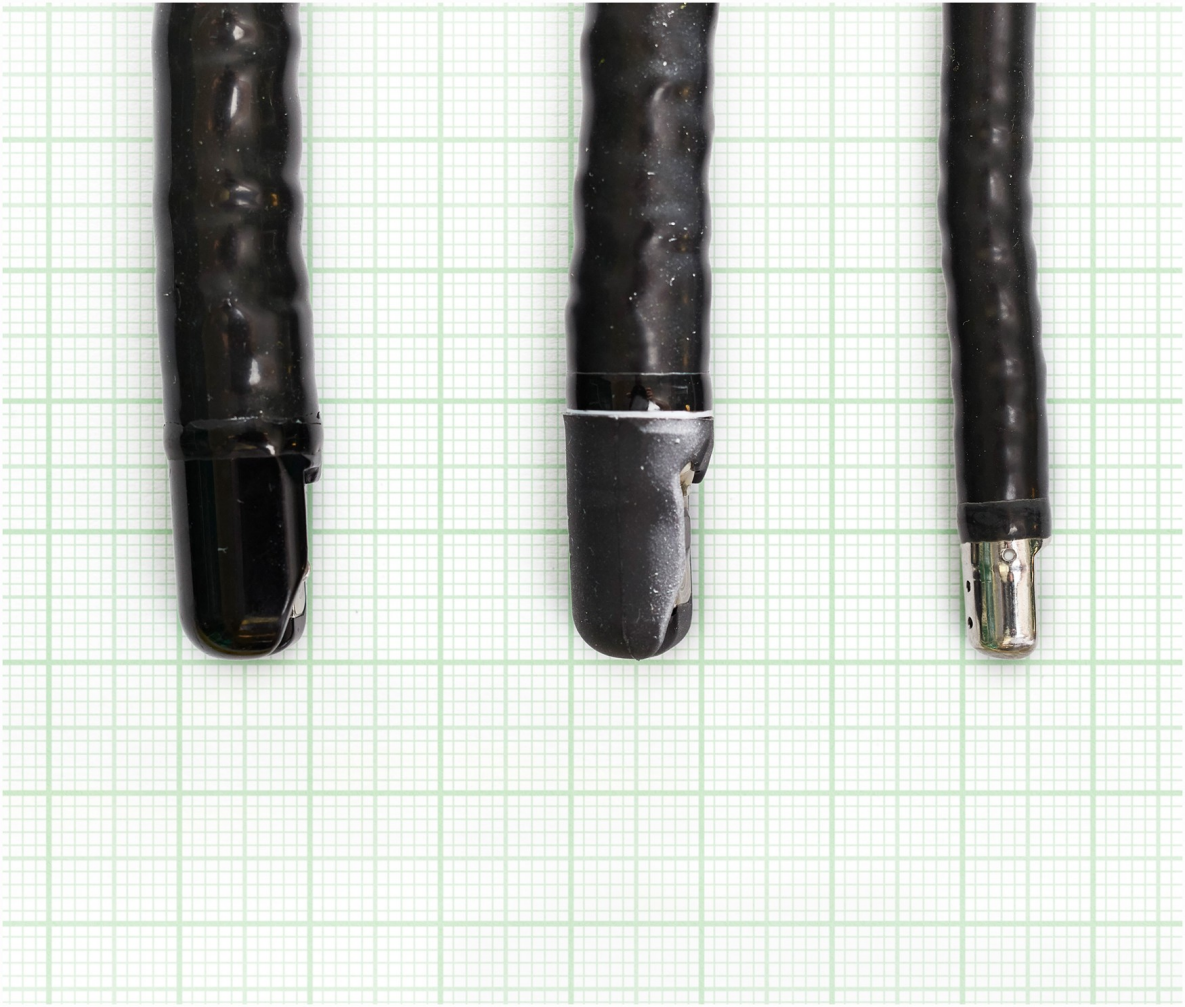
Olympus duodenoscopes: Therapeutic duodenoscope TJF-160 VR (left), diagnostic duodenoscope JF-140R (in the middle), pediatric duodenoscope Olympus PJF (right).

In children aged 1–3 years, a diagnostic duodenoscope (JF-140R, Olympus) was used, with an outer diameter of 11 mm and a working channel of 3.2 mm. In children older than 3 years, a therapeutic lateroscope (TJF-160 VR), with an outer diameter of 13.5 mm and working channel of 4.2 mm, was used Fig 1.

In patients with enough space in the duodenum, precut sphincterotomes from Olympus, (KD-17Q-1) and sphincterotomes (KD-19Q-1), or triple lumen sphincterotomes (FlowCut KD-301Q-0725) were used in combination with a standard 0,035 inch (0,89 mm) guide wire.

The procedure was done with the patient in the left lateral position. The endoscope was inserted into the second portion of the duodenum and positioned in front of the papilla. The patient was then moved to the prone position. Serum amylase levels, liver enzyme levels, and blood counts were assessed in all patients 6 h and 18 h after the investigation.

## Results

During a 20-year period, 856 ERCPs were performed in 626 pediatric patients. There were 294 boys and 332 girls. The median age of the children was 4 years and 11 months (range 12 days to 17 years and 6 months), and 58.8% of ERCP procedures were therapeutic and 41.2% were diagnostic.

ERCP was done with a pediatric duodenoscope (PJF) in 240 procedures, an adult diagnostic duodenoscope (JF140R) in 226 procedures, and an adult therapeutic duodenoscope (TJF 160 VR) in 390 procedures. Regarding ERCPs where the pediatric duodenoscope was used, patients’ weight ranged from 1.4 kg to 12 kg, and their ages ranged from 14 days to 13 months.

The children were stratified into four age groups; <1 year, 1–6 years, 7–12 years, and 13–19 years. The indications for ERCP stratified by age are reported in Table 1.

**Table 1:** The indications for ERCP stratified by age.

Indication/ age (years)	<1	1–6	7–12	13–19
Biliary obstruction	223	142	118	189
Chronic pancreatitis	2	20	61	58
Pancreatic duct disruption	0	7	9	9
Bile leak	0	7	5	6

### Successful procedures

**Fig 2:**
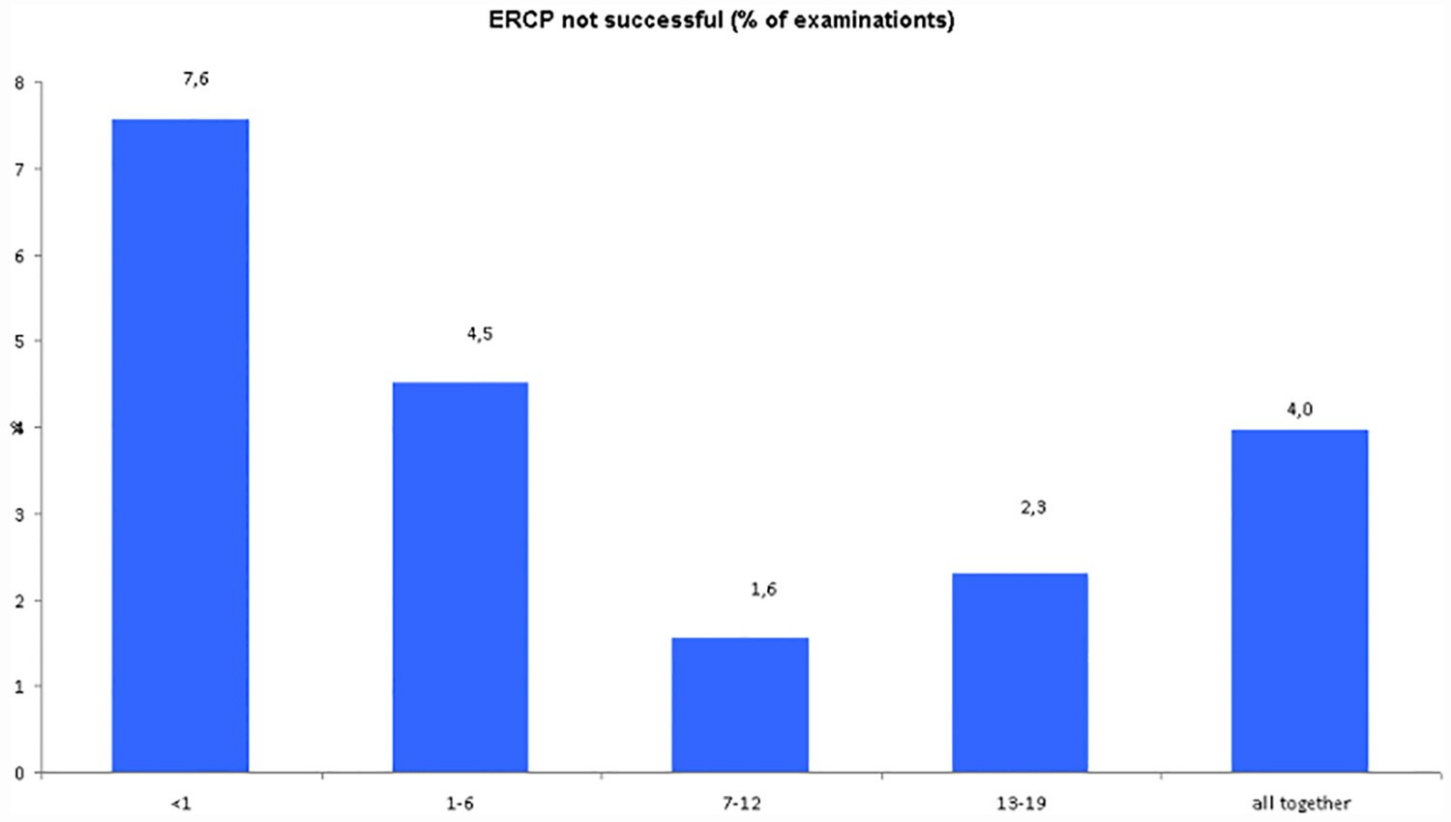
ERCP not successful (%).

Primary ERCP was successfully completed in 593 of 627 patients (94,6%), having cannulation and completion of the desired endoscopic therapy. Repeated ERCP in patients with previous sphincteroromy was 100% successful. Some patient had more than one procedure after papilotomy (for example stent exchange). Details are listed in Table 2 and Fig 2. In one case PTC was used after unsuccessful ERCP drainage in a girl with primary sclerosing cholangoitis and stenosis of the common bile duct.

**Table 2:** ERCP not successful.

Age (years)	Number of		ERCP not successful		
	Patints	ERCP	patiets	% of patienst	% of examinations
<1	219	225	17	7,76	7,56
1–6	130	177	8	6,15	4,52
7–12	119	193	3	2,52	1,55
13–19	159	261	6	3,77	2,30
all together	627	856	34	5,42	3,97

We did not use the PTCD rendezvous technique—in this case the guide wire did not pass through the stenosis to the duodenum. For endoscopic ultrasound (EUS) assisted technique the diameter of the EUS was in this case too large.

The assessment of ERCP success between age groups was statistically analyzed using Chi-square test, Kruskal-Wallis test and Dunn's multiple comparisons test. There was no significant difference between age groups.

### Diagnostic findings

**Fig 3:**
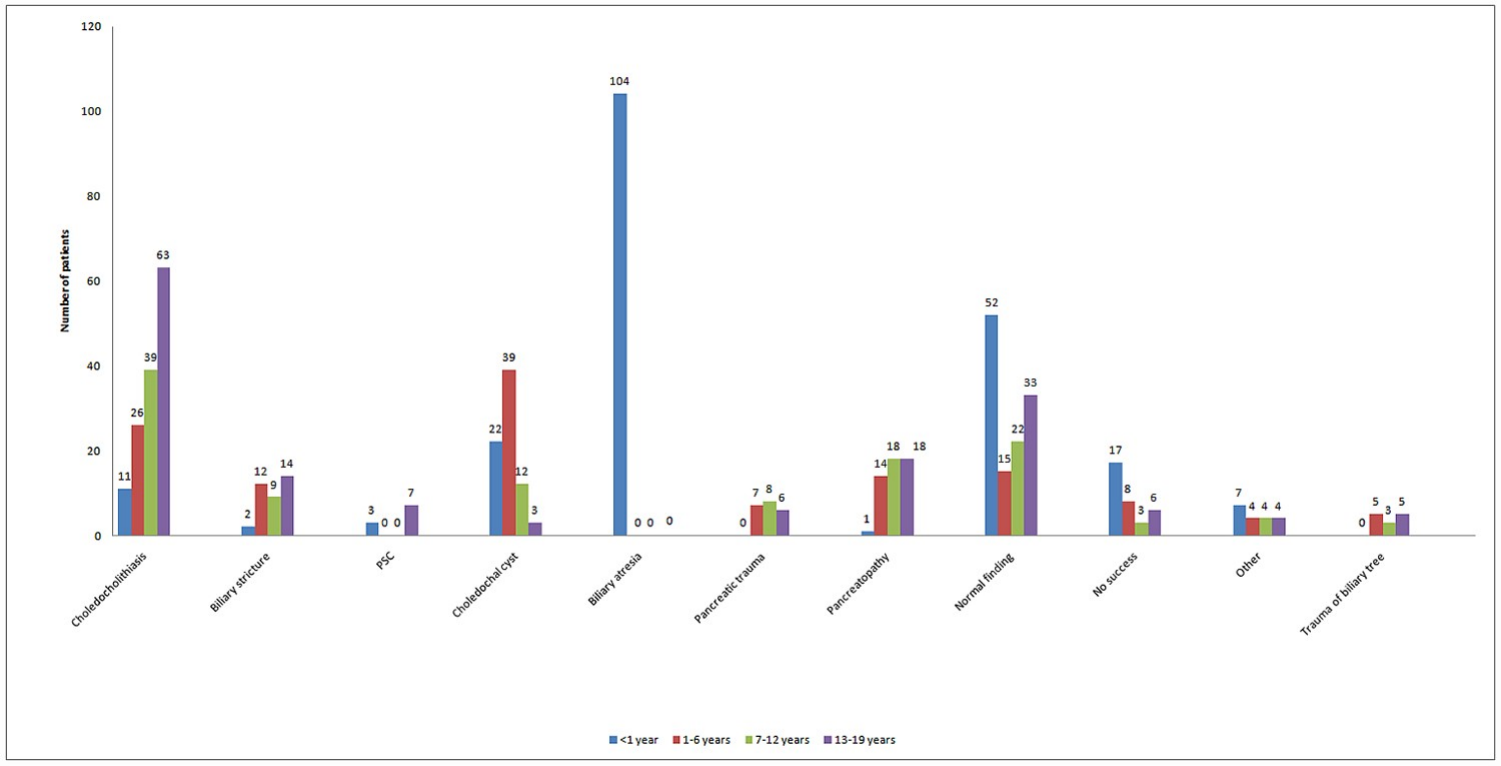
Diagnostic findings stratified by age.

Diagnostic findings were divided into eleven categories. The incidences of each finding were then divided into age groups, with significant differences observed. The different frequencies of cholelithiasis, biliary strictures, PSC, choledochal cysts, biliary atresia, and pancreatic or biliary traumatic ruptures in each of the age groups are shown in Fig 3.

### Endoscopic therapy

Therapeutic procedures were done in 368 patients (58.8% of patients). The therapeutic procedures were sphincterotomy of the biliary duct (195 procedures, 31.1% of patients, 22.8% of procedures), sphincterotomy of the pancreatic duct (25 procedures, 4% of patients, 2.9% of procedures), insertion of biliary drains (241 procedures, 38.4% of ptients, 28.2% of procedures), insertion of pancreatic drains (78 procedures, 12.4% of patients, 9.1% of procedures), extraction of biliary stones (113 procedures, 18% of patients, 13.2% of procedures), and extraction of pancreatic stones (10 procedures, 1.6% of patients, 1.2% of procedures) (Table 3).

**Table 3:** Therapeutic procedures.

Therapeutic procedures	Number of procedures	% of patients	% of procedures
Biliary duct sphincterotomy	195	31,10	22,78
Biliary dreinage insertion	241	38,44	28,15
Biliary stones extraction	113	18,02	13,20
Pancreatic duct sphincterotomy	25	3,99	2,92
Pancreatic dreinage insertion	78	12,44	9,11
Pancreatic stones extraction	10	1,59	1,17

The therapeutic procedure was done in 10.2% of ERCP (23 ERCP) in age group under one year, 70,6% of ERCP (125 ERCP) in age group 1–6, 72.5% of ERCP (140 ERCP) in age group 7–12 and 73.6% of ERCP (192 ERCP) in age group 13–19 years.

Insertion of biliary stents in children aged less than 1 year was done without sphincterotomy. Sphincterotomy was done only in one child under one year, in age eleven months. The frequency of endoscopic therapeutic procedures varied over time, without any positive or negative trend.

**Fig 4:**
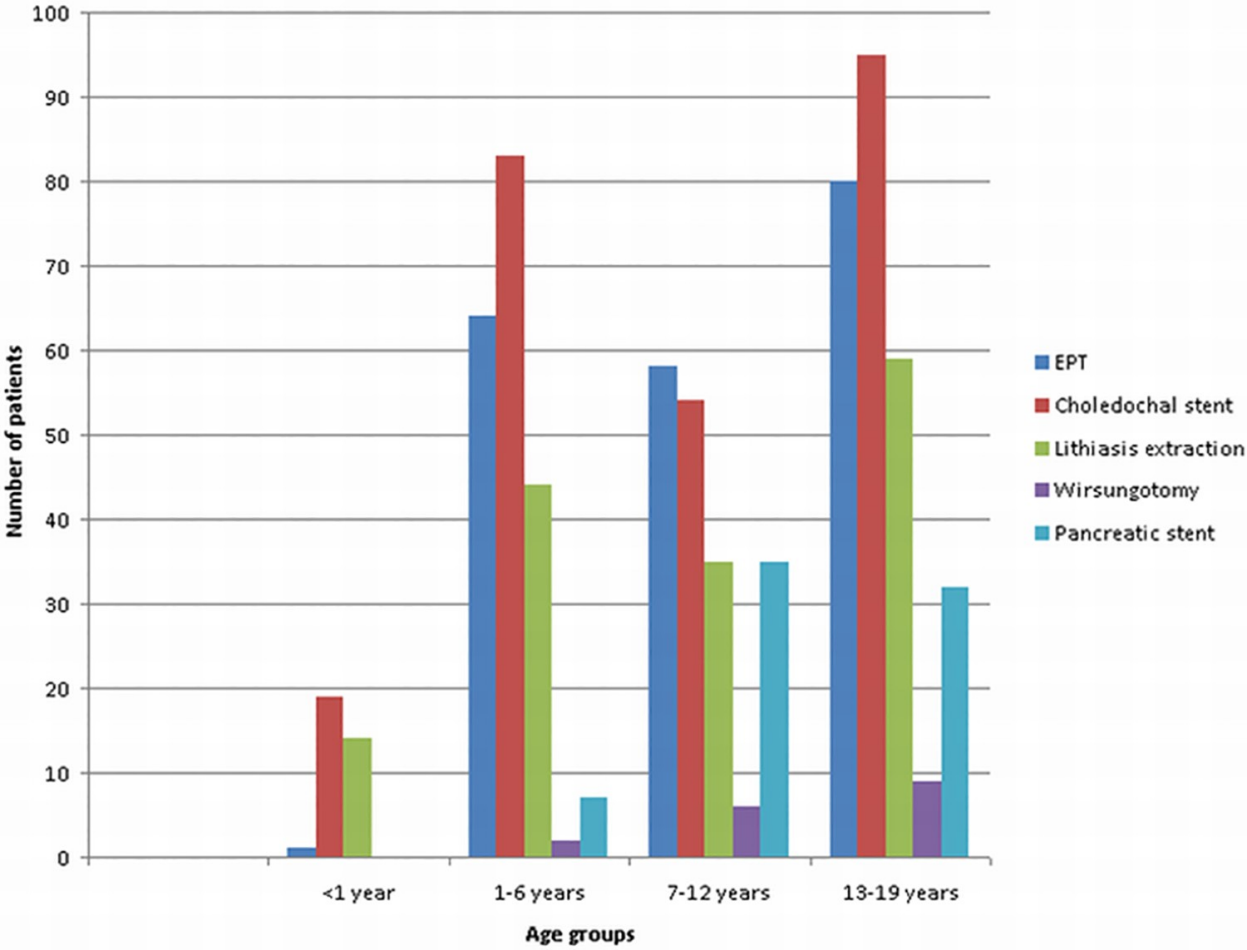
Endoscopic therapeutic procedures stratified by age—Absolute numbers.

**Fig 5:**
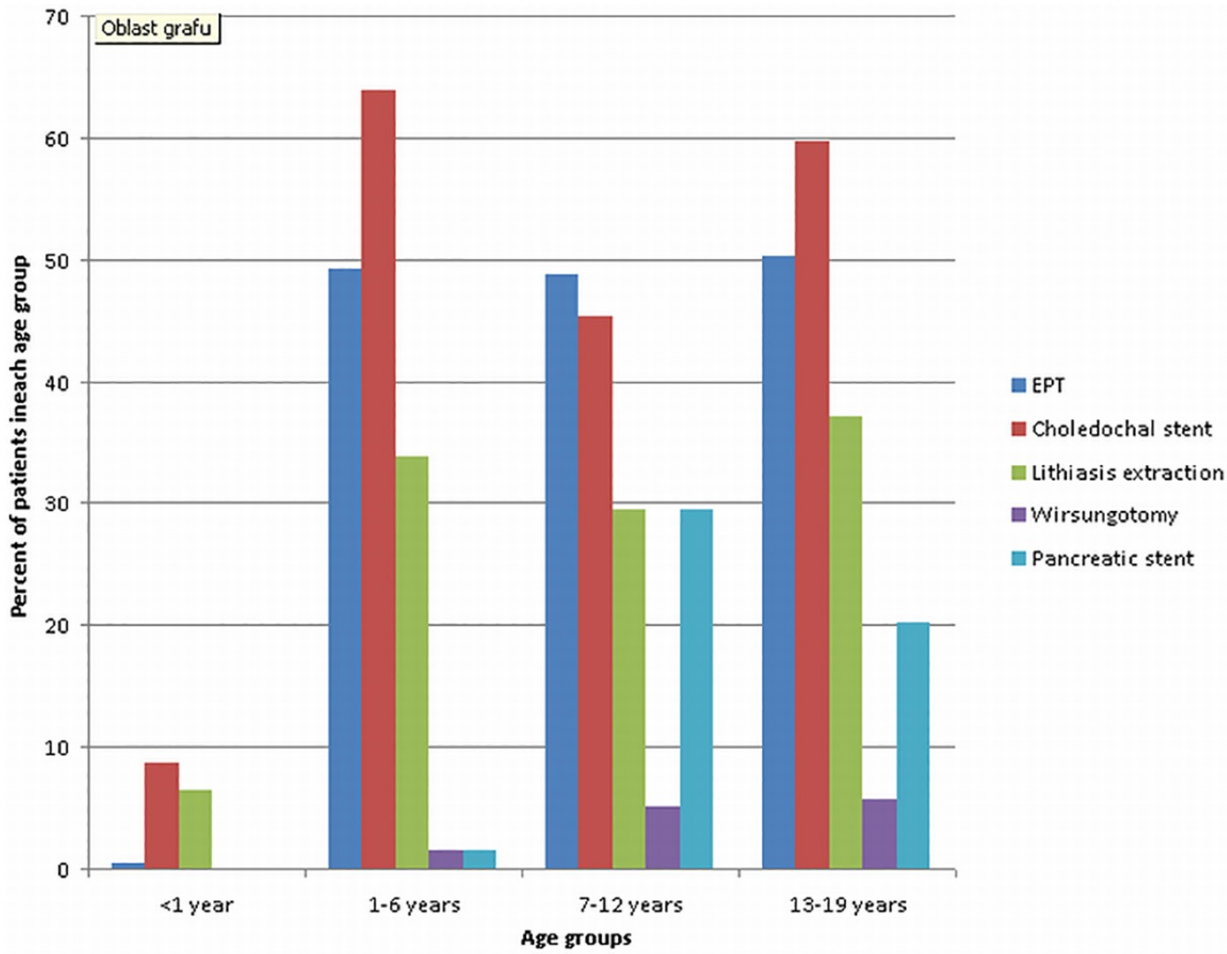
Percentage of endoscopic therapeutic procedures in each age group.

The age stratification of therapeutic procedures are in Table 4 and Fig 4. In the under one year age group choledochal stent placement is the most common procedure. The higher count of stent insertion in age groups 1–6 and 7–12 is due to repeated stent changes in some patients. Distribution of therapeutic procedures in each age group is clearly shown in Fig 5.

**Table 4:** Endoscopic therapeutic procedures stratified by age—Absolute numbers.

Age (years)	<1 year	1–6 years	7–12 years	13–19 years	Total
EPT	1	64	58	80	203
Choledochal stent	19	83	54	95	251
Lithiasis extraction	14	44	35	59	152
Wirsungotomy	0	2	6	9	17
Pancreatic stent	0	7	35	32	74

### Complications

Clinical follow up was done for 48 hours after the procedure in all patients, together with routine investigations of blood count, serum amylase, and lipases eight hours after the procedure. Distribution of complications in age groups is shown in Table 5. In 62 cases (7.2% of ERCP procedures), elevated serum lipase and amylase was observed without clinical manifestation of pancreatitis. The highest rate of this complication is in age group under one year (13.8% of ERCP procedures). This was statistically significant (p = 0,0018 using Chi-square test). In patients with confirmed biliary atresia we can see elevated amylases in 30% of ERCP procedures. Nobody in the group under one year had acute pancreatitis. In 10 cases (age7-12 years two cases, age 12–19 eight cases), mild pancreatitis (fever, elevation of amylase and lipase, and abdominal pain) was observed. In the age group 13–19 years 2 cases of acute hemorrhage after papilla sphincterotomy was observed; both cases were resolved with endoscopic therapy (hemostatic clips). In one case, delayed sphincterotomy bleeding with melena developed 24 hours after the procedure. The complication was resolved with an acute endoscopic intervention and hemostatic clips.

**Table 5:** Distribution of complications in age groups.

Age group	<1	1–6	7–12	13–19	all together
ERCP	225	177	193	261	856
Elevated amylase	31	8	11	12	62
% to ERCP	13.78	4.52	5.70	4.60	7.24
Midl pancreatits	0	0	2	8	10
% to ERCP	0	0	1.04	3.07	1.17
Bleeding after EPT	0	0	0	2	2
% to ERCP	0	0	0	0.77	0.23
Cholangoitis	0	1	1	1	3
% to ERCP	0	0.56	0.52	0.38	0.35
Septic complications after insertion of a pancreatic stent	0	0	0	1	1
% to ERCP	0	0	0	0.38	0.44
Suspicion of post ERCP perforation	1	0	0	0	1
% to ERCP	0.44	0	0	0	0.44
Retroperitoneal depot of contrast medium	1	0	0	0	1
% to ERCP	0.04	0	0	0	0.04
All together	33	9	14	24	80
% to ERCP	14.7	5.1	7.3	9.2	9.35

Clinical signs of cholangitis developed after inserting stents in three cases (age groups 1–6 years one case, 7–12 years one case, 12–19 one case). In all these cases, the cholangitis was resolved with stent exchange and intravenous ATB therapy. In one case (age group 12–19 years), septic complications (fever, elevation of C-reactive protein) developed after insertion of a pancreatic stent in a patient with chronic pancreatitis and pancreaticolithiasis. This case was resolved by exchanging the stent and administration of intravenous ATB therapy. In one case with suspicion of perforation (abdominal pain, elevation of CRP) in a cholestatic infant (age group 0–1 year) with a choledochal cyst after an unsuccessful biliary stent insertion, the problem was resolved conservatively with parenteral nutrition and intravenous antibiotic therapy. In one child (age group 0–1 year) a retroperitoneal depot of contrast medium emerged during procedure. It was resolved conservatively (parenteral feeding, antibiotic therapy). There were no cases of ERCP-induced necrotic pancreatitis. No mortality was observed after ERCP.

## Discussion

Our study demonstrates the different uses of diagnostic and therapeutic ERCP in pediatric patients. The procedures were performed by gastroenterologists who mostly treat adult patients. Primary ERCP was successfully completed in 94.6% patients, it is comparable to rates reported in other studies. [12, 13]

We also wanted to demonstrate the different roles of ERCP in different age groups. This is apparent in the different number of diagnostic and therapeutic procedures done in the different age groups. In the group of children aged under 1 year, the main role of ERCP was in confirming or excluding biliary atresia and pancreaticobiliary maljunction. The newborns were indicated for neonatal cholestasis. The sensitivity of ERCP in the diagnosis of biliary atresia is 86%, with a specificity of 94%.[2] Patients with normal findings are those in whom ERCP excluded the suspicion of biliary atresia. The second most frequent pathological diagnosis was choledochal cyst in patients who are indicated for obstructive jaundice. Typical of such cases is the relatively small dilatation of the bile duct, with plugs stemming from protein debris from the wall of the bile duct. In these cases, it is necessary to insert a stent and prepare the child for a later operation at an older age. ERCP has recently been shown to be superior to MRCP for visualization of the main pancreatic duct in pancreaticobiliary maljunction.[14] In the past, newborns who had suspicion for biliary athresia were indicated for surgery (Kasai procedure) with laparotomy and peroperative cholangiography. However, some patients (23.1%) are found to have intact bile ducts, rendering the operation as unnecessary. Our previous data published in Endoscopy 2010 [2]–shows that ERCP is specifically useful for avoiding unnecessary Kasai procedures in infants with suspicion of biliary atresia.

The third most frequent pathological diagnosis was choledocholithiasis. The therapeutic role of ERCP is limited in children aged less than one year for two reasons. First, pediatric duodenoscope from Olympus cannot be used for papilla sphincterotomy; therefore, choledocholithiasis must be resolved by insertion of a biliary stent without papilla sphincterotomy. All children were treated successfully with the combination of an inserted 5 fr stent and conservative treatment with ursodeoxycholic acid. After 12 months of age, the stent can be extracted and papilla sphincterotomy can be done, with extraction of the choledocholithiasis.

In the group of children aged 1 to 6 years, the most frequent diagnosis was choledochal cyst, and the second most frequent diagnosis was choledocholithiasis.

In children aged between 1–3 years, it was necessary to use a diagnostic duodenoscope (JF140R, Olympus) with an outer diameter of 11 mm and a working channel of 3.2 mm. The smaller outer diameter gives the endoscopist more maneuverability in the duodenum, and more space before the papilla of Vateri. The therapeutic procedures (precut, papilla sphincterotomy and insertion of biliary and pancreatic stents) can be done, though with some difficulty due to limited space in front of the papilla Vateri. In such cases, the pediatric double lumen sphincterotome with an outer diameter of 1.75 mm (Medi-Globe RotaCut GSP-21-17-020) was used. With this working channel, it is possible to insert only 7 fr polyethylene stents. On the other hand, the smaller diameter of the inserted stents in bile duct stenosis was not associated with clotting of the stents. This is probably caused by the lower lithogenic index of bile in children. [15,16] The therapeutic duodenoscope, TJF 160 VR, with a 13.5-mm outer diameter and a 4.2-mm working channel, was used in children older than 3 years. This enables dilatation of benign biliary strictures with stents of a larger diameter.

The lowest success rate was in the youngest group of children. This lower success rate was not statistically significant. ERCP failure in infants was mostly the consequence of the very narrow offering of endoscopic eqipment for this field. Failure of duodenoscope intubation was the main problem of the children aged 12–16 months where we have tried to use duodenoscope with an outer diameter 11 mm for therapeutic procedure.

In children aged 7 to 12 years, the most frequent diagnosis was choledocholithiasis. Its frequency increases with age and absolutely dominates in the group aged 13–19 years. Pathologies of the pancreas were the second most frequent indications in both groups. The frequency of traumatic damage to the biliary tract or pancreas was most common in those aged 7 to 19 years.

From our data, different frequencies of pathological findings in different age groups in the biliary and pancreatic tract are visible. The most frequent diagnosis in the youngest age group was biliary atresia. Choledochal cysts are most frequent in children aged 1 to 6 years, while the incidence rapidly declined in the older age groups. The incidence of benign bile duct stenosis also increased with age.

Our study demonstrates the consistent quantity of therapeutic ERCP during the observation period (1999–2018). This is in opposition to the increasing trend described in the United States between the years 2000–2009.[11] The overall complication rate found in this study was similar to rates observed in adult populations. Post procedural pancreatitis was found in 1.6% of cases, which is lower than the 5–7% incidence typically reported in adults.[17]

ERCP was used as a diagnostic tool at the beginning of our observation period. Thanks to the development of imaging methods, especially MRCP, diagnostic indications have decreased. In recent years and especially the last five years ERCP has been indicated only as a therapeutic procedure when therapeutic intervention is expected. The only one exception from this rule are newborns with suspected biliary atresia.

With the increasing incidence of childhood obesity in central Europe, the incidence of choledocholithiasis in children will also increase. In our study, the main indication was choledocholithiasis and most likely, the need for therapeutic ERCP in the future will increase as well.[18]

## Conclusion

The results of our retrospective study illustrate the efficacy and safety of diagnostic and therapeutic ERCP in a large series of infants and children, with technical success and complication rates that are comparable to those in adults. We also demonstrate the different roles of ERCP in different age groups.

Differences were observed in the specific indications and different frequencies of diagnosis in different age groups. In children aged under1 year, the main role of ERCP was in confirming or excluding the diagnosis of biliary atresia. ERCP is specifically useful for avoiding unnecessary Kasai procedures in infants with suspicion of biliary atresia. The therapeutic role of ERCP in this group is technically limited. In children aged 1 to 6 years, the most frequent diagnosis was choledochal cyst, and the second most frequent diagnosis was choledocholithiasis. In this group, the role of ERCP is mainly therapeutic. In children aged 7 to 12 years and 13 to 19 years, the most frequent diagnosis was choledocholithiasis and pancreatic pathology. The therapeutic potential of ERCP plays a key role in these age groups.

Despite their effectiveness and the importance of their role in performance of endoscopic procedures, the production of diagnostic duodenoscope JF140R from Olympus was stopped in the year 2015; additionally, production of pediatric duodenoscope Olympus PJF was stopped in the year 2013. If the production of these endoscopes is not restored, the diagnostic and therapeutic role of ERCP in children under 36 months is at risk because the diameter of the TJF series endoscopes does not allow for a safe introduction of the device into the duodenum.

## Supporting information

S1 Table Diagnostic finding divided into eleven categories stratified by age. (DOC)

## References

[1] Giefer MJ, Kozarek R A. Technical outcomes and complications of pediatric ERCP. *Surgical Endoscopy* 2015:29:3543–3550. 10.1007/s00464-015-4105-1 25673350

[2] Keil R, Snajdauf J, Rygl M, Pycha K, Kotalova R, Drabek J, et al Diagnostic efficacy of ERCP in cholestatic infants and neonates–a retrospective study on a large series. *Endoscopy*, 2010:42(02), 121–126. 20140829 10.1055/s-0029-1215372

[3] Keil R, Drabek J, Lochmannova J, Stovicek J, Rygl M, Snajdauf J, et al What is the role of endoscopic retrograde cholangiopancreatography in assessing traumatic rupture of the pancreatic in children? *Scandinavian journal of gastroenterology* 2016:51 2: 218–224. 10.3109/00365521.2015.1070899 26200695

[4] Keil R, Drabek J, Lochmannova J, Stovicek J, Rygl M, Snajdauf J,. et al Endoscopic treatment of bile duct post-traumatic and post-operative lesions in children. *Scandinavian journal of gastroenterology* 2017:52 8: 870–875 10.1080/00365521.2017.1309453 28388849

[5] Drabek J, Keil R, Stovicek J, Lochmannova J, Hlava S, Snajdauf J, et al The role of endoscopic retrograde cholangiopancreatography in choledochal cysts and/or abnormal pancreatobiliary junction in children. *Przegląd Gastroenterologiczny* 2017: 12 4: 303 10.5114/pg.2017.72107 29359001 PMC5771456

[6] Enestvedt BK, Tofani C, Lee DY, Shah PM, Ginsberg GG, Long WB, et al 792 ERCP in the Pediatric Population Is Successful and Efficacious.*Gastrointestinal Endoscopy*, 2012: 75 4: AB164–AB165.

[7] Troendle DM, Liu QY, Kim KM, & Barth B 853 ERCP in younger vs older children: initial report from the multicenter pediatric ERCP database initiative. *Gastrointestinal Endoscopy*, 2015: 81 5: AB173.

[8] Otto AK, Neal MD, Slivka AN, & Kane TD An appraisal of endoscopic retrograde cholangiopancreatography (ERCP) for pancreaticobiliary disease in children: our institutional experience in 231 cases. *Surgical endoscopy*, 2011: 25 8: 2536–2540. 10.1007/s00464-011-1582-8 21359895

[9] Limketkai BN, Chandrasekhara V, Kalloo AN & Okolo PI Comparison of performance and safety of endoscopic retrograde cholangiopancreatography across pediatric age groups. *Digestive diseases and sciences*, 2013: 58 9: 2653–2660. 10.1007/s10620-013-2691-0 23709156

[10] Felux J, Sturm E, Busch A, Zerabruck E, Graepler F, Stüker D, et al ERCP in infants, children and adolescents is feasible and safe: results from a tertiary care center. *United European gastroenterology journal*, 2017: 5 7: 1024–1029. 10.1177/2050640616687868 29163969 PMC5676540

[11] Pant C, Sferra TJ, Barth BA, Deshpande A, Minocha A, Qureshi WA, et al Trends in endoscopic retrograde cholangiopancreatography in children within the United States, 2000–2009. *Journal of pediatric gastroenterology and nutrition*, 2014: 59 1: 57–60. 10.1097/MPG.0000000000000333 24509307

[12] Halvorson L, Halsey K, Darwin P, & Goldberg E The safety and efficacy of therapeutic ERCP in the pediatric population performed by adult gastroenterologists. *Digestive diseases and sciences*, 2013: 58 12: 3611–3619. 10.1007/s10620-013-2857-9 24026405

[13] Paris C, Bejjani J, Beaunoyer M, & Ouimet A Endoscopic retrograde cholangiopancreatography is useful and safe in children. *Journal of pediatric surgery*, 2010: 45 5: 938–942. 10.1016/j.jpedsurg.2010.02.009 20438931

[14] Hiramatsu T, Itoh A, Kawashima H, Ohno E, Itoh Y, Sugimoto H et al Usefulness and safety of endoscopic retrograde cholangiopancreatography in children with pancreaticobiliary maljunction. *Journal of pediatric surgery*, 2015: 50 3: 377–381. 10.1016/j.jpedsurg.2014.08.024 25746692

[15] Bergmann KV., Becker M, & Leiss O Biliary cholesterol saturation in non-obese women and non-obese men before and after puberty. *European journal of clinical investigation*, 1986: 16 6: 531–535. 3104054 10.1111/j.1365-2362.1986.tb02173.x

[16] Halpern Z, Vinograd Z, Laufer H, Gilat T, Moskowitz M, & Bujanover Y Characteristics of gallbladder bile of infants and children. *Journal of pediatric gastroenterology and nutrition*, 1996: 23 2: 147–150. 8856581 10.1097/00005176-199608000-00009

[17] Cohen S, Bacon BR, Berlin JA, Fleischer D, Hecht GA, Loehrer PJ et al. National institute of health state-of-the-science conference statemenet: ERCP for the diagnosis and therapy January 14–16 2002. 2002: p. 803–9. 10.1067/mge.2002.129875 12447289

[18] Kobzova J, Vignerova J, Blaha P, Krejcovsky L & Riedlová The 6th nationwide anthropological survey of children and adolescents in the Czech Republic in 2001. Cent Eur J Public Health. 2004;12(3):126–30 15508410

