# Supplementary material for: ERCP in infants, children, and adolescents—Different roles of the methods in different age groups
Source: PLoS One. 2019 Jan 17;14(1):e0210805. doi: 10.1371/journal.pone.0210805 (PMC6336232; doi:10.1371/journal.pone.0210805)
Supplement: S1 Table — (DOC) [file pone.0210805.s001.doc]

| Age (years) | | <1 | 1-6 | 7-12 | 13-19 | Total |
| --- | --- | --- | --- | --- | --- | --- |
| Pts in group | | 219 | 130 | 119 | 159 | 627 |
| Choledocholithiasis | Pts | 11 | 26 | 39 | 63 | 139 |
|  | % | 5,0 | 20,0 | 32,8 | 39,6 | 22,2 |
| Biliary stricture | Pts | 2 | 12 | 9 | 14 | 37 |
|  | % | 0,9 | 9,2 | 7,6 | 8,8 | 5,9 |
| PSC | Pts | 3 | 0 | 0 | 7 | 10 |
|  | % | 1,4 | 0,0 | 0,0 | 4,4 | 1,6 |
| Choledochal cyst | Pts | 22 | 39 | 12 | 3 | 76 |
|  | % | 10,0 | 30,0 | 10,1 | 1,9 | 12,1 |
| Biliary atresia | Pts | 104 | 0 | 0 | 0 | 104 |
|  | % | 47,5 | 0,0 | 0,0 | 0,0 | 16,6 |
| Pancreatic trauma | Pts | 0 | 7 | 8 | 6 | 21 |
|  | % | 0,0 | 5,4 | 6,7 | 3,8 | 3,3 |
| Pancreatopathy | Pts | 1 | 14 | 18 | 18 | 51 |
|  | % | 0,5 | 10,8 | 15,1 | 11,3 | 8,1 |
| Normal finding | Pts | 52 | 15 | 22 | 33 | 122 |
|  | % | 23,7 | 11,5 | 18,5 | 20,8 | 19,5 |
| No success | Pts | 17 | 8 | 3 | 6 | 34 |
|  | % | 7,8 | 6,2 | 2,5 | 3,8 | 5,4 |
| Other | Pts | 7 | 4 | 4 | 4 | 19 |
|  | % | 3,2 | 3,1 | 3,4 | 2,5 | 3,0 |
| Trauma of biliary tree | Pts | 0 | 5 | 3 | 5 | 13 |
|  | % | 0,0 | 3,8 | 2,5 | 3,1 | 2,1 |
